# Early mortality risk in incident Chinese hemodialysis patients: a retrospective cohort study

**DOI:** 10.1080/0886022X.2017.1337583

**Published:** 2017-06-21

**Authors:** Xinju Zhao, Mei Wang, Li Zuo

**Affiliations:** Department of Nephrology, Peking University People’s Hospital, Beijing, China

**Keywords:** Hemodialysis, incidence, mortality rate, end-stage renal disease

## Abstract

**Background:** Early mortality risk of maintenance hemodialysis (MHD) patients varies by country and ethnicity. Here, early mortality in incident Chinese HD patients were studied.

**Methods:** Data from 1 January 2007 to 31 December 2013 were pulled from Beijing dialysis registry system. All included patients were followed to the end of 2013. This time period of dialysis was divided into six intervals (≤120, 121–365 days; 1–2, 2–3, 3–4, ≥5 years). Patients’ demographics, primary cause of end-stage renal disease (ESRD), date of first HD, date of death, cause for death, date and cause of censoring were extracted from the registry database. All-cause mortality (per 100 patient-years) was calculated for each period stratified by sex, age and cause of ESRD. Monthly mortality rates were also calculated.

**Results:** A total of 11,955 patients were included, 6738 were males and 5217 were females. The mean age at dialysis initiation was 57.7 ± 16.1 years. The median follow-up time was 19.8 months. There were total 2555 deaths. The overall mortality rate was 8.2 per 100 patient-years. Mortality rates were 18.7, 7.5, 6.9, 6.9, 6.5 and 6.2 in each period. The first 2 months mortality rates were 41.9 and 16.6 per 100 patient-years. Higher mortality was observed in patients who were older, female, diabetic and hypertensive.

**Conclusions:** The most critical period was the first 2 months of dialysis initiation. Patients who were older, female, diabetic and hypertensive had higher risk of early mortality. Our analysis highlighted that the transitional period from sever CKD stages to dialysis initiation, when optimal supportive care should be adopted, was crucial for patients’ survival.

## Introduction

Hemodialysis (HD) therapy is a life-saving and life-sustaining procedure that improves the life expectancy of patients with end-stage renal disease (ESRD). However, the adjusted rates of all-cause mortality were 6.5–7.9 times greater for dialysis patients than for individuals in the general population [1]. We previously reported that the crude mortality rate for maintenance hemodialysis (MHD) patients in Beijing was much lower than that in the United States [2]. However, in our previous analysis, only included stable patients who had been on MHD for ≥90 days. Incident patients usually experience a high risk of mortality during the first few months of dialysis initiation, which is generally defined as the risk of early mortality [1,3–6].

Previous studies have shown that early mortality rates and patterns varied remarkably among different countries and ethnic groups. The United States Renal Data Services (USRDS) 2013 annual report showed that in the first year of HD, all-cause mortality peaked in the second month [1]. In 2010, the all-cause mortality for incident HD patients was 44 per 100 patient-years in the second month and gradually fell to 20.1 in month 12. Study using DaVita data in the United States showed the mortality rate was 47 per 100 patient-years within the first 3 month after HD initiation and 30 per 100 patient-years within the first year [3]. Another report compared the unadjusted risk of mortality (expressed as percentage) within the first 90 days and in the entire first year after dialysis initiation for incident MHD patients in three large renal registries (USRDS patients in 2006, Canadian Organ Replacement Register data in 2006, and European Renal Association–European Dialysis and Transplant Association Registry data between 2004 and 2008). The mortality rates were 5.6–8.6% in the first 90 days and 16.2–24.3% in the first year [4]. Ninety days after dialysis initiation was usually considered the critical period. Recently, Dialysis Outcomes and Practice Patterns Study (DOPPS) reported that the mortality rate within 120 days after HD initiation was 26.7 per 100 patient-years, with a peak within the first month (29.3 per 100 patient-years) [5]. In the DOPPS study, early mortality rates from 11 countries were compared and Japan was the only country from East Asia. There is no report in literature about early mortality risk in Chinese population and China has always been considered as the biggest developing country in East Asia. Therefore, we studied the early mortality rate and pattern in Beijing where a relatively complete HD registry is in place.

## Materials and methods

Beijing Hemodialysis Quality Control and Improvement Center (BJHDQCIC) was set up by the Beijing Department of Health in 2003 and one of its main missions was to establish and maintain ESRD registry [2]. Currently, all 110 hemodialysis facilities in Beijing are managed by BJHDQCIC, except 10 military hemodialysis facilities. Before 2007, only facility-level data were collected at the end of every year. From 2007, BJHDQCIC started collecting patient-level data using an electrical data capturing system.

The study protocol was approved by the Medicine Ethics Committee of the Peking University People’s Hospital.

### Study population

All the incident HD patients in BJHDQCIC registry from 1 January 2007 to 31 December 2013 were included in the analysis. Patients with previous dialysis history and transferred to Beijing during this period were excluded. Patients were censored if they met one of the criteria listed below during the follow-up: (1) patients were transferred to dialysis facility outside of Beijing; (2) patients abandoning dialysis treatment; (3) patients switched to peritoneal dialysis; (4) patients had recovery of kidney function and stopped dialysis treatment; (5) patients received kidney transplant. All the included patients were followed to the end of 2013 or until death, lost follow-up. Death of a patient was ascertained either by information provided by the immediate family members or by reviewing the death certificate. All patients were >18 years, as defined by the registry enrollment criteria. For each patient, the demographics, primary cause of ESRD, date of HD initiation, date of death, cause for death, date and cause of censoring were extracted from the registry database. The information was anonymized prior to analysis.

### Statistical analysis

Baseline characteristics at HD initiation were reported as the mean and standard deviation (SD) for continuous variables and percentage for categorical variables. Continuous variables not normally distributed were also presented as median and interquartile range (IQR). Crude mortality rates were estimated as the number of deaths per 100 patient-years. The 95% confidence intervals (CI) were estimated using Byar’s method [7].

The main variable of interest was time on dialysis, categorized as Period (P) 1 (≤120 days), P2 (121–365 days), P3 (1–2 years), P4 (2–3 years), P5 (3–4 years) and P6 (≥5 years). We adopted the same definition of early dialysis period (≤120 days) as used in the DOPPS study. The primary outcome was all-cause mortality, analyzed with time-dependent Cox regression model, stratified by study phase and adjusted for sex, age and primary cause of ESRD. Patients’ age at the start of dialysis were divided into five categories: <45, 45 − 54, 55–64, 65–74 and ≥75 years. The primary cause of ESRD included diabetes (DM), hypertension (HT), chronic glomerulonephritis (CGN), other known causes (chronic interstitial nephritis, urinary tract infection, polycystic kidney disease, tumor, etc.) and unknown causes. Using P2 as the reference category, similar to the DOPPS study, adjusted hazard ratios (HR) for the other intervals were calculated.

The first 360 days after dialysis initiation was divided into 12 periods (30 days per period), overall mortality, mortality by primary cause of ESRD (DM vs. other) and age (<65 y or ≥65 y) were also calculated, respectively.

Statistical significance was defined as *p* < .05. All statistical analyses were performed with SAS, version 9.3 (SAS Institute, Cary, NC).

## Results

During the 6-year study period, 11,955 patients were enrolled and 2253 patients censored. There were 6738 males (56.4%) and 5217 females (43.6%). The mean age was 57.7 ± 16.1 years (range 18.0–99.5 y). The average age was 55.9 ± 16.5 for male and 60.0 ± 15.3 for female. Patients’ demographics were shown in Table 1. Diabetes as the cause of ESRD accounted for 29.5% of patients. The reasons for censoring were listed as Table 2.

**Table 1.** Demographics of Beijing incident MHD patients.

	Beijing cohort	Death number	Patient-days	Mortality rate*
Number of patients	11,955	2555	11,407,904	8.2
Age (mean, *m*)	57.7 ± 16.1			
<45	2626 (22.0%)	201	2,494,485	2.9
45–54	2371 (19.8%)	354	2,483,682	5.2
55–64	2602 (21.8%)	537	2,614,048	7.5
65–74	2422 (20.3%)	698	2,357,842	10.8
>75	1934 (16.2%)	765	1,457,847	19.2
Gender				
Male	6738 (56.4%)	1371	6,313,693	7.9
Female	5217 (43.6%)	1184	5,094,211	8.5
Cause of ESRD				
Diabetes	3531 (29.5%)	952	3,435,179	10.1
Nondiabetic	8424 (70.5%)	1603	7,972,725	7.3
CGN	3220 (26.9%)	420	3,525,690	4.3
HT	2282 (19.1%)	485	2,141,809	8.3
Unknown	1768 (14.8%)	447	1,324,648	12.3
Other causes	1154 (9.7%)	251	980,578	9.3
Study phases				
≤120 days	11,955	643	1,254,955	18.7
121–365 days	9927	473	2,314,280	7.5
>365 days	9065	1439	7,838,669	6.7
1–2y	9065	539	2,856,053	6.9
2–3y	6614	389	2,052,944	6.9
3–4y	4692	256	1,436,674	6.5
≥5y	3170	255	1,492,998	6.2

Mortality rate*: death number per 100 patient-years.

MHD: maintenance hemodialysis; y: years old; CGN: chronic glomerulonephritis; HT: hypertension.

**Table 2.** Reasons for censoring.

Reasons for censoring	Censored number
Transfer out of dialysis facility in Beijing	370
Abandoning dialysis treatment	145
Switch to peritoneal dialysis	213
Recovery of kidney function	843
Kidney transplant	682
Total	2253

The median follow-up time was 19.8 months (IQR 5.7, 40.6 months). There were a total of 2555 deaths occurred and 7147 patients remained in the study at the end of 2013. Of these, 643, 473, 539, 389, 256 and 255 deaths occurred in period 1–6, respectively. The overall mortality rate was 8.2 per 100 patient-years (95% CI: 7.1, 9.4). Mortality rates in each period were listed in Table 1. Mortality in the first 120 days of dialysis initiation constituted 57.6% of the mortality occurred in the first year and 25.2% of all death in this study.

**Figure 1.**
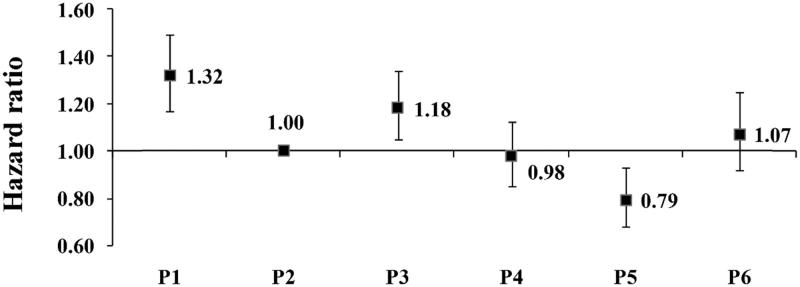
Adjusted hazard ratios (HRs) by time on dialysis. HRs were adjusted for sex, age and primary cause of ESRD. P(Period) 1 ≤ 120 days; P2 121–365 days; P3 1–2 years; P4 2–3 years; P5 3–4 years; P6 ≥ 5 years.

Using P2 as the reference, the adjusted HRs for the other periods in this cohort was shown as Figure 1. The difference of HRs between periods 1–6 was statistically significant (*p* < .001).

**Figure 2.**
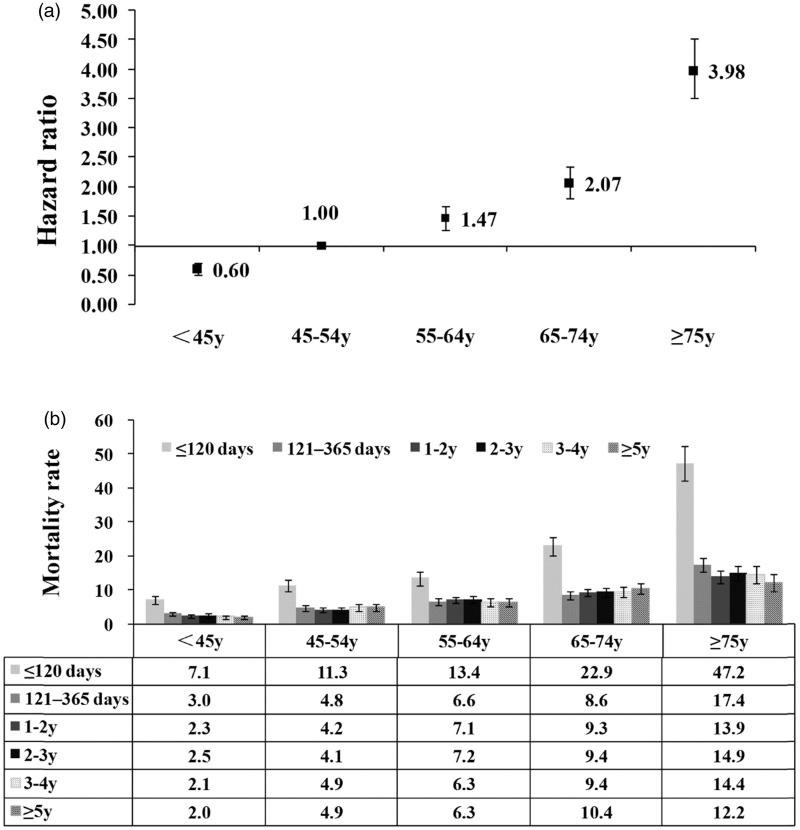
Mortality by age and time on dialysis. (a) The overall mortality rates increased with age. (b) Mortality rates decreased after the early period in each age group. Mortality rate: number of deaths per 100 patient-years. Error bars correspond to 95% confidence intervals calculated using the Byar’s approximation. y: years.

The overall mortality rates gradually increased with age (*p* < .001, Table 1). For people ≥65 y, the overall mortality rate was 14.0/100 patient-years (95% CI: 11.7, 16.6). Compared to patients 45–54 years old, the risks of mortality for other age groups all increased (Figure 2(a)) (*p* < .001). In each age group, the mortality rate at early stage of dialysis was the highest (Figure 2(b)).

**Figure 3.**
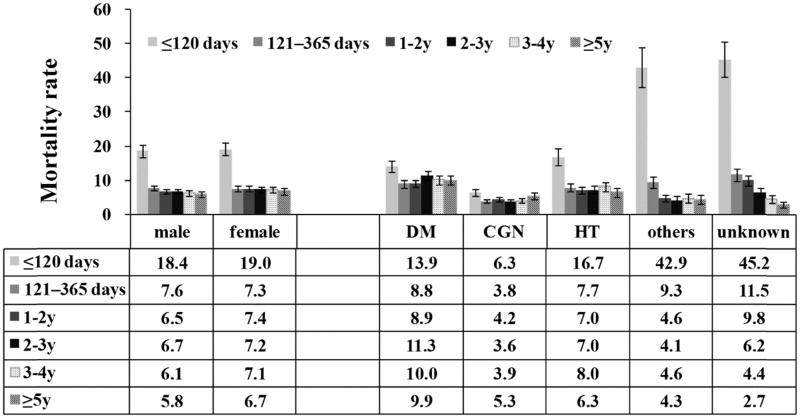
Mortality rates by gender and cause of ESRD. Mortality rate: number of deaths per 100 patient-years. Error bars correspond to 95% confidence intervals calculated using the Byar’s approximation. CGN: chronic glomerulonephritis; DM: diabetes; HT: hypertension; y: years.

The overall mortality rates for men and women were 7.9 and 8.5 per 100 patient-years, respectively, adjusted HR = 1.05 (95% CI: 0.97, 1.14, *p* = .23), (Figure 3).

We stratified patients according to their primary cause of ESRD. Generally, the mortality rates were 10.1 in diabetic patients and 7.3 in nondiabetic (NDM) patients. The mortality rate was lowest in CGN group, followed by diabetic group and highest in other and unknown causes group (Figure 3).

**Figure 4.**
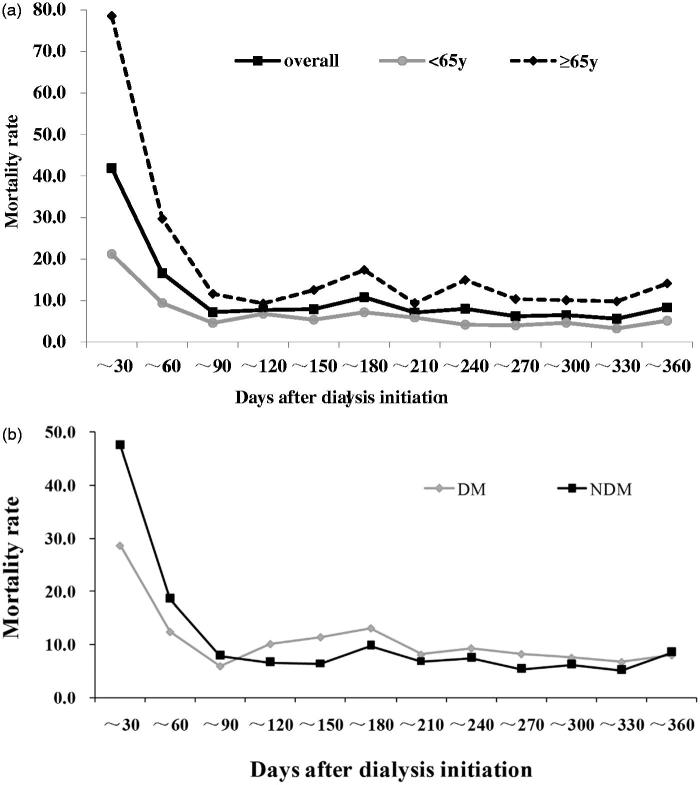
Mortality rates by dialysis vintage, age and primary cause. *y* coordinate mortality rate (per 100 patient-years); *x* coordinate: days after dialysis initiation. (a) The monthly mortality rates and mortality rates by age (<65 y or ≥65 y) were calculated. The first 30-day mortality was highest in our cohort. Mortality rate for elderly patients (≥65 y) was higher in every interval than younger patients (<65 y). (b) Within the 90 days of dialysis initiation, mortality rate for NDM patients was higher than DM patients, while the trend reversed after that. DM: diabetes; NDM: nondiabetic disease; y: years old.

Furthermore, we also estimated crude mortality rates by month. The overall mortality, mortality by primary cause of ESRD and age group were shown in Figure 4(a,b). In our cohort, the highest mortality risk was in the first month after dialysis initiation, then the risk dropped dramatically to a lower level and became stable after 90 days into dialysis. The trend of mortality for patients <65 y and ≥65-y old was similar. NDM patients had higher mortality risk than DM patients in the first 90 days as well.

## Discussion

The study showed that the most critical period for incident HD patients was within the first two months after dialysis initiation with the mortality rate being the highest in the first month. After the early period, the mortality rate dropped substantially and stayed in a stable low level.

Our results were basically consistent with previous researches mentioned earlier. However, we had the highest mortality rate in the first month, while the highest mortality rate was in the second month both in the USRDS 2013 annual report (44 per 100 patient-years) and Foley’s research (also used USRDS data). Hence, Foley et al. questioned the inadequate registry data collection, especially in the early stage [8]. In Foley’s study, they calculated weekly mortality rates in the first year of treatment for HD patients from USRDS (2005–2009) and found that mortality rate peaked at 37.0 per 100 person-years in week 6 and declined steadily to 14.8 by week 51 [8]. We had a relatively similar early mortality rate. But, we have a much lower first-year mortality rate.

As for the high early mortality rate, we speculated that there might be a number of reasons. (i) Inadequate predialysis nephrology care could be a very important factor [3–6,8–14]. A systematic review [13] showed patients could benefit from early nephrology referral even in CKD stage by reducing mortality rate up to 40–50%. The most recent study suggested that adequacy of predialysis nephrology care should consider both the timing of the nephrology referral and the number and timing of visits [14]. However, in China, surveillance and follow-up care for CKD was just implemented and systemic predialysis nephrology care was unavailable for many CKD patients even in Beijing. (ii) Social–economic condition may also play an important role. If patients could not afford the optimal treatment, they might be at higher risk of early death. However, since medical insurance covers most HD-related care in Beijing, and patients pay very little for HD treatment and related medications, we suspect that social–economic condition might not contribute much to the high early mortality in our study. (iii) Cultural and public health policy may partially account for the different mortality pattern among countries [4,6]. For example, in certain counties, old and ill patients may be more likely to receive conservative care rather than dialysis [15]. In China, many ESRD patients who were very ill and had poor prognosis still insisted on receiving dialysis treatment instead of ‘conservative kidney management’. They were at very high risk of death shortly after dialysis initiation. The current regulations do not allow doctors to provide ‘conservative care’ without the family member’s consent. These differences in culture and regulations might contribute to the high early mortality in our study. (iv) ‘Selection bias’ may be another reason. Our analysis included all HD centers facilities in Beijing area, while in other studies, dialysis sites agreeing to participate may have better performance on average [6]. Besides, in some countries, hospital HD centers treating sicker patients may be small in size and not be chosen for study participation. Thus, the mortality can be underestimated. Goodkin noted this possibility [16], and speculated that this might partially account for the data discrepancy in Japan where the annual mortality rate among Japanese DOPPS patients was lower than that reported by the Japanese Society of Dialysis Therapy (JSDT) [17]. (v) Our registry system was designed for ESRD patients only and excluded patients with acute kidney injury or acute renal failure. However, there was a possibility that patients with acute kidney injury or acute renal failure were included in this analysis. In our analysis, we censored 843 patients whose renal function recovered during the follow-up. While the misclassification was only possible in the group whose primary cause of ESRD was unknown or missing (14.8%), it could result in an overestimation of mortality rate. We conducted sensitivity analysis limited to only patients with known cause for ESRD and received the similar results (not shown in this research).

As mentioned earlier, appropriate selection of conservative management or dialysis for frail elderly patients with advanced kidney disease is of immense importance. These patients often suffer loss of functional status, impaired quality of life and increased mortality after dialysis initiation [18,19]. Nephrology clinicians should provide patient-centered care for them and help them and their caregivers to decide if the potential benefits of dialysis outweigh the risks. Some decision-making framework had been proposed [20]. However, there is rare validated integrated procedure to follow. Experts pointed out that ‘Current paradigms of care for this highly vulnerable population are variable, prognostic and assessment tools are limited and quality of care, particularly regarding conservative and palliative care, is suboptimal.’ in KDIGO Controversies Conference on Supportive Care in Chronic Kidney Disease [21]. And we should also notice that there is variation in ethics, culture and physician perception about this issue [22,23]. In China, only conservative kidney management without dialysis for those frail elderly patients is not feasible now due to ethical and cultural concerns.

We also had a lower overall mortality rate compared with that in the previous DOPPS study, which was 15.0 per 100 patient-years. Although as discussed previously, the overall mortality rate in our study could have been underestimated, it may not fully explain the significant difference between this study and the DOPPS study, due to several reasons: (i) The finding that we had high first month mortality rate suggested that the missing number of death could not be large, or else we would not have gotten so many death records; (ii) the overall mortality rate in the DOPPS study was almost twice of that in our study. If we had similar overall mortality rate, the death number should be doubled, and obviously the missing records could not reach that number. On the contrary, the DOPPS study underestimated mortality rate for incident patients by including both incident and prevalent dialysis patients in the analysis. As shown in USRDS 2013 annual report, the mortality rate for prevalent patients could be much lower than that for incident patients [1]. From these aspects, the mortality rate in Beijing was truly lower than that in the DOPPS study. Age might also be an influential factor. Patients in our study were generally younger than those in the DOPPS study. However, we also analyzed patients whose age were ≥65 years old, and the overall mortality rate was still lower than that in the DOPPS study. Other potential factors including timing of dialysis initiation, the vascular access type (catheter or fistula), and serum indicators could not be further analyzed here.

We conducted subgroup analysis by the cause of ESRD. Lower risk in CGN group and higher risk in diabetic group were not unexpected. Among the three common causes for ESRD (CGN, DM, HT), the mortality risk in HT group was the highest.

Although not statistically different, our study found that the mortality rates for females tended to be higher than those for males in most dialysis periods. This was inconsistent with the common knowledge that generally females survive better than males. In our study, the average age of female was 4 years older than male. Older age is definitely an important factor for increased mortality [5,6]. Although our analysis stratified by age, there may still be residual confounding associated with age.

The main limitation of our study was its retrospective design, limited data collection from a registry system established only for a large metropolitan area. There could be other unmeasured factors associated with mortality that were not accounted for. We also have some other limitations in this analysis. The mortality risk in this critical period (first two months) could be underestimated. In Beijing, the participation into BJHDQCIC registry was not mandatory. For short-term dialysis patients, especially those who died soon after initiating dialysis there was a possibility that they were not captured by the registry system as questioned by Foley et al. [8]. We faced the same issue in this database as well.

Results in this study were important and complements to our previous findings of prevalent HD patients [2]. Different findings on mortality pattern from other countries were also important to understand the ESRD population globally. Our analysis highlighted that the transitional period from sever CKD stages to initiating dialysis was crucial for patients’ survival. Inclusion of palliative and supportive care for advanced CKD is a patient-centered strategy that should be adopted appropriately and timely. Patients who were older female, diabetic and hypertensive might need more attention from healthcare providers, as they had relatively higher early mortality risk in our study.
